# LncRNA GNAS-AS1 facilitates ER^+^ breast cancer cells progression by promoting M2 macrophage polarization via regulating miR-433-3p/GATA3 axis

**DOI:** 10.1042/BSR20200626

**Published:** 2020-06-30

**Authors:** Shi-Qin Liu, Zhi-Yang Zhou, Xue Dong, Lei Guo, Ke-Jing Zhang

**Affiliations:** Department of General Surgery, Xiangya Hospital, Central South University, Changsha 410008, Hunan Province, P.R. China

**Keywords:** ER+ Breast cancer, GATA3, GNAS-AS1, Macrophage polarization, miR-433-3p

## Abstract

**Objective:** ER^+^ breast cancer is the most common type of breast cancer, which seriously affects the physical and mental health of women. Recently, lncRNAs mediated tumor-associated macrophages (TAM) were identified to involve in tumorigenesis. Therefore, the present study aimed at demonstrating the regulatory network of GNAS-AS1 in TAM-mediated ER^+^ breast cancer progress.

**Methods**: The expression levels of genes were evaluated using qRT-PCR. The proportions of polarized macrophages (M1, M2) were assessed by flow cytometry. Cell proliferation, migration and invasion were evaluated by CCK-8, wound healing and transwell assay, respectively. Double-luciferase reporter system was used to detect the interaction between molecules. Western blot was applied to test protein levels.

**Results**: The expression of GNAS-AS1 was obviously increased in ER^+^ breast cancer tissues and cell lines, as well as M2 macrophages. GNAS-AS1 facilitated the capabilities of proliferation, migration and invasion of ER^+^ breast cancer cells by accelerating M2 macrophage polarization via directly sponging miR-433-3p. GATA3, as a target of miR-433-3p, could positively regulate by GNAS-AS1. Furthermore, either miR-433-3p overexpression or GATA3 knockdown impaired the effects of GNAS-AS1 on M2 macrophage polarization and ER^+^ breast cancer cells progression.

**Conclusion**: GNAS-AS1/miR-433-3p/GATA3 axis promoted proliferation, metastasis of ER^+^ breast cancer cells by accelerating M2 macrophage polarization. The mechanism may provide a new strategy and target for ER^+^ breast cancer treatment.

## Introduction

Breast cancer, as a malignant tumor that seriously affects the survival and life of women, is known as the first killer of women [1]. Current evidences have suggested that the incidence and mortality rate of breast cancer are significant increase, with an estimated 278,800 new cases and 64,600 deaths in China, although the dramatic advances in diagnosis and treatment [2]. ER^+^ breast cancer is the most common subtype of this disease, which accounts for nearly 75% of total [3]. The treatment options for ER^+^ breast cancer includes surgery, chemotherapy, radiation therapy, molecular targeted therapy, immunotherapy and so on [4]. However, current clinical treatment has been challenged due to the therapeutic resistant of patients, which caused by the molecular heterogeneity and complicated biology process of ER^+^ breast cancer [5]. Therefore, to explore the molecular mechanisms underlying ER^+^ breast cancer progression may provide great value for searching novel therapeutic strategies and potential targets of breast cancer.

Macrophages, a class of high plastic cells, can polarize into M1 or M2 subtypes in response to the surrounding microenvironment [6]. It’s different from M1 macrophages, M2 macrophages play anti-inflammatory effects through producing anti-inflammatory cytokines such as interleukin (IL)-10 and transforming growth factor (TGF)-β, which is linked to immunosuppression and tumorigenesis [7,8]. Macrophages are dominating immune cell population in tumor microenvironment (TME), and its heterogeneity is the main feature of the TME [9]. M2 macrophages, one subtype of tumor-associated macrophages (TAM), have an abundant level in breast cancer and could stimulated tumor growth, metastasis, matrix degradation and angiogenesis [10]. A clinical study focused on breast cancer revealed that the high density of CD204-positive TAM predicted worse clinical prognosis, including recurrence-free survival, distant recurrence-free survival and breast cancer-specific survival [11]. However, little is known about the molecular mechanism behind the macrophage polarization process in ER^+^ breast cancer.

Long noncoding RNAs (lncRNAs), with length more than 200 nucleotides, are defined as endogenous RNAs, which exerted crucial regulatory roles in multiple physiological processes, such as apoptosis, angiogenesis and inflammation [12]. Growing evidences have proved that lncRNAs are involved in the modulation of M2 macrophage polarization [13]. GNAS antisense (GNAS-AS1) is one of the alternative transcripts of human *GNAS* locus localized on chromosome 20q13.3 [14]. As a novel lncRNA, the roles of GNAS-AS1 in biological and pathogenic processes remain poorly understood. Early investigation has demonstrated that GNAS-AS1 enhanced M2 macrophage polarization to promote the migration and invasion of non-small cell lung cancer (NSCLC) cells [15]. However, this regulatory network of GNAS-AS1 in ER^+^ breast cancer still not well-studied.

In the present study, our results illustrated that GNAS-AS1 was dramatically up-regulated in M2 macrophages, ER^+^ breast cancer cells and clinical tumor tissues. In addition, overexpressing GNAS-AS1 elevated M2 macrophage polarization and promoted the capabilities of proliferation, migration and invasion of ER^+^ breast cancer cells through directly repressing miR-433-3p, which could target GATA3 to markedly inhibit its expression. These results unveiled a novel regulation network of GNAS-AS1 in TME and breast cancer progression, which might provide some new useful references for ER^+^ breast cancer.

## Material and methods

### Clinical tissue collection

Clinical ER^+^ breast cancer tissues and adjacent non-cancer tissues were surgically collected from 20 patients with ER^+^ breast cancer in Department of Breast Surgery, Xiangya Hospital, Central South University (Changsha, China) from February 2017 to November 2018. The specimens were divided into small pieces, and kept in freezer for further experiments. Informed consents from each breast cancer patient were obtained before operation. The study including tissue collection was approved by the Ethical Committee of Xiangya Hospital, Changsha, China, and followed institutional ethical guidelines.

### Reagents

Antibodies to GATA3 (Cat.5852) and GAPDH (Cat.5174) were obtained from Cell Signaling Technology. Human interferon (IFN)-γ, IL-4 and M-CSF were purchased from Thermo Fisher Scientfic, and lipopolysaccharides (LPS) was purchased from Solarbio.

### Cell culture

Human ER^+^ breast cancer cell lines (MCF-7, T47D), normal mammary epithelial cell line (MCF10A) and human monocytes (THP-1) were obtained from the Chinese Academy of Sciences Institute (Shanghai, China). The cells were maintained in RPMI 1640 medium (THP-1) (Hyclone, America), MEGM medium (MCF10A) (Hyclone, America) and DMEM/F-12 (T47D, MCF-7) (Gibco, America), supplemented with 10% (v/v) fetal bovine serum (FBS, Gibco, America) and 1% penicillin/streptomycin (Beyotime Biotechnology, China). Primary monocytes were maintained in RPMI1640 medium supplemented with 10% (v/v) FCS (Gibco, America), 2 mM glutamine (Sigma, G6392) and1% antibiotic–antimycotic in humidified atmosphere at 37°C with 5% CO_2_.

### Macrophage polarization and culture

Human peripheral blood mononuclear cells (PBMCs) from the blood of healthy donors were separated using Ficoll and Percoll density gradient centrifugations as described previously [16]. Human monocytes were sorted form PBMCs using CD14 and CD11b^+^ monoclonal antibodies (Thermo Fisher, America), and then CD14^+^ and CD11b^+^ monocytes were maintained in RPMI medium with 10% FBS at 37°C and 5% CO_2_ in ultra-low attachment flasks for 7 days, followed by treatment with 50 ng/ml M-CSF for 6 day. For macrophage polarization, M1 polarization was induced by treatment with 100 ng/ml LPS and 100 ng/ml IFN-γ for 24 h. M2 polarization was induced by treatment with 20 ng/ml IL-4 for overnight. M0 cells were collected after 48 h of incubation with serum-free medium.

### Real-time quantitative PCR (qRT-PCR)

According to the manufacturer’s instructions, total RNA was extracted by using TRIzol reagent (Invitrogen, U.S.A.), and used for first-strand complementary DNA synthesis with PrimeScript™ RT reagent Kit (TaKaRa, China) following the detection of RNA concentration. qRT-PCR was performed by using SYBR Green mix (TaKaRa, China) in triplicate in ABI 7500 Fast Real-Time PCR System (Applied Biosystems, U.S.A.) to quantify the levels of gene transcripts. Relative quantity of gene expressions were normalized to GAPDH or U6, and finally calculated by the standard 2^−△△Ct^ method. All the primers were purchased from Sangon Biotech and their sequences were listed in Table 1.

**Table 1:** Primer sequences information in qRT-PCR assay

Primer names	Sequences (5′-3′)
GNAS-AS1 Forward	GACGCCTTTCCTACGG
GNAS-AS1 Reverse	TGGTAACGCACCTTCG
GAPDH Forward	GGAGCGAGATCCCTCCAAAAT
GAPDH Reverse	GGCTGTTGTCATACTTCTCATGG
GATA3 Forward	TCGTCCTCCTCCTTGTCGG
GATA3 Reverse	GGAAGGTGAAGAGGTGCGG
TNF-α Forward	TGTTCCTCAGCCTCTTCTCCT
TNF-α Reverse	TGCAGCGGCGAAGAGCGTG
IL-6 Forward	GAGGAAGATTCCAAAGATGT
IL-6 Reverse	GGATGTACCGAATTTGTCA
IL-10 Forward	AGCACTGCTCTGTTGCCTG
IL-10 Reverse	GTGCAGCTGTTCTCAGACTG
Arginase 1 Forward	GTATTGAGAAAGGCTGGTCTG
Arginase 1 Reverse	TCAAGCAGACCAGCCAAACAC
miR-433-3p Forward	GGTGAGCCTGTCATTATTG
miR-433-3p Reverse	GCATGTCAGTCGTGCAGT
U6 Forward	GGTGCTCGCTTCGGCAGCACA
U6 Reverse	TTGTGCAGGGTCCGAGGT

### THP-1-differentiated macrophage

For macrophage differentiation, THP-1 cells were cultured in RPMI 1640 medium with 150 nM PMA (Sigma, #P1585) and 10% FBS for 48 h.

### Plasmids and cell infection

The human cDNA was used as the template to amplify the segment of GNAS-AS1 by PCR. The forward primer was 5′-CTAGAATTCTAGGGGGCGCCGCGTT-3′; the reverse primer was 5′-CTAGGATCCTTGACAGGGTGCATCTGG-3′. The segment of GNAS-AS1 was cloned into the restrictive sites of the pSin-vector to construct the overexpression plasmid (pSin-GNAS-AS1). Then, lipofectamine 2000 (Thermo Fisher, America) was used to transfect the plasmids into THP-1-differentiated macrophages for further experiments according to the manufacturer’s instructions. For GNAS-AS1, miR-433-3p and GATA3 knockdown, cells were transfected with 20 nM small interfering RNAs against GNAS-AS1, GATA3 or 50 nM miR-433-3p inhibitor (Sangon, Shanghai) using lipofectamine 2000, respectively. Non-targeting siRNA (si-NC), empty vector (pSin-NC) and inhibitor NC were used as control.

### Cell viability

Cell viability was evaluated in the present study by using a Cell Counting Kit-8 (Solarbio, China) following the manufacturer’s instructions. Briefly, MCF-7 and T47D cells were plated in the lower chambers, and co-cultured with the THP-1-differentiated macrophages (under different treatments) in the upper chambers at a ratio of approximately 10:1 for 48 h. The chambers were placed into the incubator, 10 μl of CCK-8 solution was added to each well at each time point (0, 24, 48, 72 h), and then incubated for 2 h at 37°C. Amicroplate reader (Perlong tech, China) was used to analyze the absorbance at 450 nm.

### Wound healing assay

Would healing assay was carried out to detect cell migration capability following designated transfection. Briefly, T47D and MCF-7 cells were plated in the lower chambers, and co-cultured with the THP-1-differentiated macrophages (under different treatments) in the upper chambers at a ratio of approximately 10:1 for 48 h. Then, T47D and MCF-7 cells were collected and then cultured in serum-free medium in plates. A straight scratch was made on the cell monolayer in each well with a sterile micropipette tip when cells grew to 90% confluent. The distance between the two edges of the wound was observed under microscopy after scratching for 24 h.

### Transwell assay

Cell invasion capabilities were detected by using transwell chambers with an 8 μm pore size membrane (Corning, U.S.A.) as previously described [17]. For invasion assay, 5 × 10^4^ T47D and MCF-7 cells were suspended in 100 μl of DMEM/F12 medium without FBS and plated into upper chambers coated with matrigel. The DMEM/F12 medium containing 10% FBS (500 μl) was added to the lower chambers. After 2 h, 5 × 10^3^ THP-1-differentiated macrophages (under different treatments) were seeding to the upper chambers, and cells were allowed to invade for 48 h under 37°C, 5% CO_2_. Non-invaded cells were removed with cotton swabs, and cells that had invaded into the lower chambers were stained with Crystal Violet solution after fixed with cold 4% paraformaldehyde for 30 min. Images were taken using an inverted microscope (Olympus, Japan), and the count of invaded cells was quantified.

### Dual-luciferase reporter assay

Briefly, the wild-type (WT) or mutant type (MUT) 3′UTR sequences of GNAS-AS1 and GATA3 were inserted into the pmirGLO-REPORT luciferase vector (Promega, Fitchburg, WI, U.S.A.), respectively. THP-1 differentiated macrophages were seeded to 24-well plates and cultured in RPMI 1640 medium supplemented with 10% FBS. When the cells grew to 80% confluent, these plasmids including GNAS-AS1-WT, GNAS-AS1- MUT, GATA3-WT and GATA3- MUT were co-transfected with miR-433-3p mimics or mimics NC into cells by using Lipofectamine 2000 (Thermo Fisher, America), respectively. After 24 h transfection, cells were collected and lysed for luciferase activity analysis as described previously [18].

### Western blot

Western blot was carried out to detect the protein level of GATA3 in THP-1 differentiated macrophages following standard methods. Briefly, cells were washed three times with PBS to remove the rest medium. Then, cells were lysed using RIPA lysis buffer (Thermo Fisher, U.S.A.) containing PMSF protease inhibitor (Thermo Fisher, U.S.A.). After quantitation by BCA protein assay kit (Thermo Fisher, U.S.A.), 30 mg proteins were electrophoresed in 10% SDS-PAGE gel (Sangon Biotech, China) and then were transferred onto PVDF membranes (Millipore, U.S.A.). Subsequently, the membranes were incubated with anti-GATA3 (1:1000, Cell Signaling Technology) and anti-GAPDH (1:2000, Santa Cruz Biotechnology) primary antibodies at 4°C for overnight and then incubated with the secondary antibody (1:2000, Cell Signaling Technology) at room temperature for 1 h. Protein levels were determined by band intensities quantitated with ECL system (Thermo Fisher, U.S.A.) and were analyzed by GraphPad Prism software.

### Statistical analysis

All statistical results were analyzed with GraphPad Prism software from three separate experiments, and presented as mean ± standard deviation (SD). Statistical significance was calculated by a two-tailed Student’s *t* test or one-way ANOVA considering *P*<0.05 as statistically significant.

## Results

### GNAS-AS1 was up-regulated in ER^+^ breast cancer, M2 macrophage and cell lines

**Figure 1:**
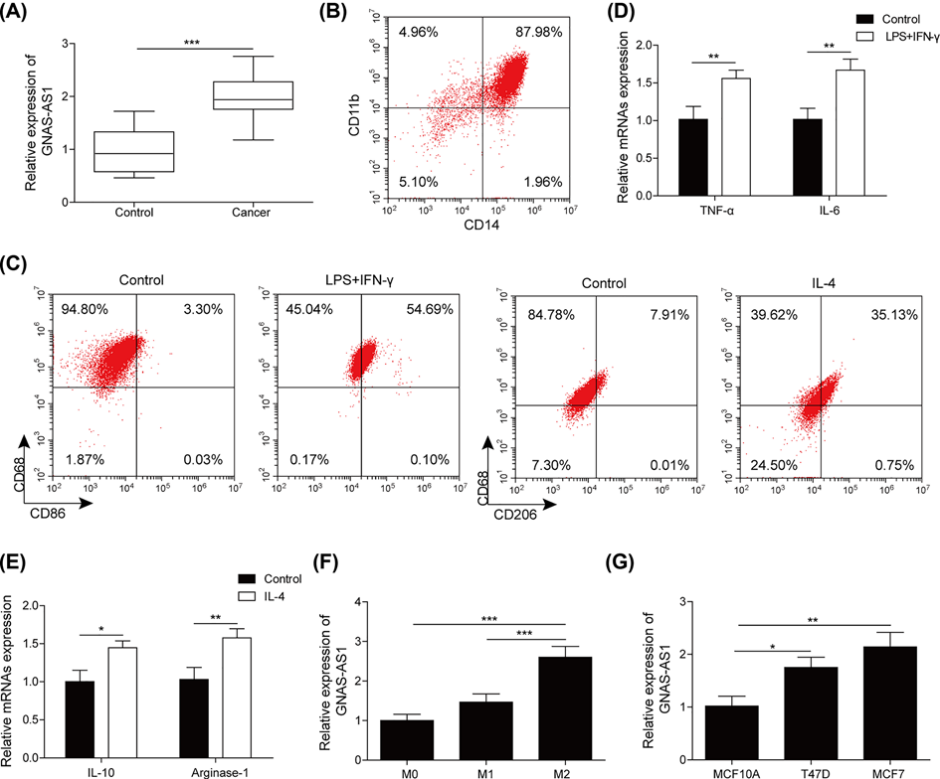
GNAS-AS1 was up-regulated in ER+ breast cancer, M2 macrophage and cell lines (**A**) The relative quantity of GNAS-AS1 in ER^+^ breast cancer tissues and adjacent normal tissues was examined by qRT-PCR. (**B**) Human monocytes were isolated from PBMCs with antibody against CD14 and CD11b and analyzed using flow cytometry. (**C**) Flow cytometry was used to quantify the proportion of M1 or M2 macrophages. (**D**) M1 macrophage markers (TNF-α, IL-6) were measured by qRT-PCR. (**E**) M2 macrophage markers (IL-10, Arginase-1) were detected by qRT-PCR. (**F**) qRT-PCR was performed to determine GNAS-AS1 expression in TAMs (M0, M1, M2). (**G**) The expression level of GNAS-AS1 in breast cancer cells (T47D and MCF-7) and normal mammary epithelial cells (MCF10A) were determined by qRT-PCR. Data with error bars are presented as the mean ± SD; *P<0.05, **P<0.01, ***P<0.001 as determined by the Student’s *t* test or one-way ANOVA test.

In the present study, cancer tissues and adjacent non-caner tissues were obtained from ER^+^ breast cancer patients to investigate the expression of GNAS-AS1. qRT-PCR assay found that GNAS-AS1 was dramatically increased in cancer tissues (Figure 1A). Given the reports of GNAS-AS1 on the regulation of M2 macrophage polarization [15], we isolated human monocytes form PBMCs using anti-CD11b and anti-CD14. As results showed in Figure 1B, human monocytes were successfully isolated. Then, human monocytes were stimulated with LPS and IFN-γ or IL-4 to induce M1 and M2 polarization, respectively [19]. Flow cytometry results were described in Figure 1C, compared with control group, LPS+IFN-γ treatment significantly enhanced CD86^+^ cells proportion, and IL-4 treatment markedly increased CD206^+^ cells proportion. Subsequently, the macrophage markers were also examined by qRT-PCR that showed that M1 macrophage markers (TNF-α and IL-6) were markedly increased after LPS+IFN-γ treatment, and M2 macrophage markers (IL-10 and Arginase-1) were dramatically elevated after IL-4 treatment (Figure 1D,E), indicating that M1 or M2 macrophages were successfully induced. Next, GNAS-AS1 expression in TAMs (M0, M1, M2) were determined using qRT-PCR, which described that GNAS-AS1 was significantly up-regulated in M2 macrophages compared with M0 or M1 macrophages (Figure 1F). In addition, GNAS-AS1 was also highly expressed in ER^+^ breast cancer cells (Figure 1G). Data from above finding demonstrated that the GNAS-AS1 may associate with the progression of ER^+^ breast cancer.

### GNAS-AS1 facilitated M2 macrophage polarization and ER^+^ breast cancer cells proliferation and metastasis

**Figure 2:**
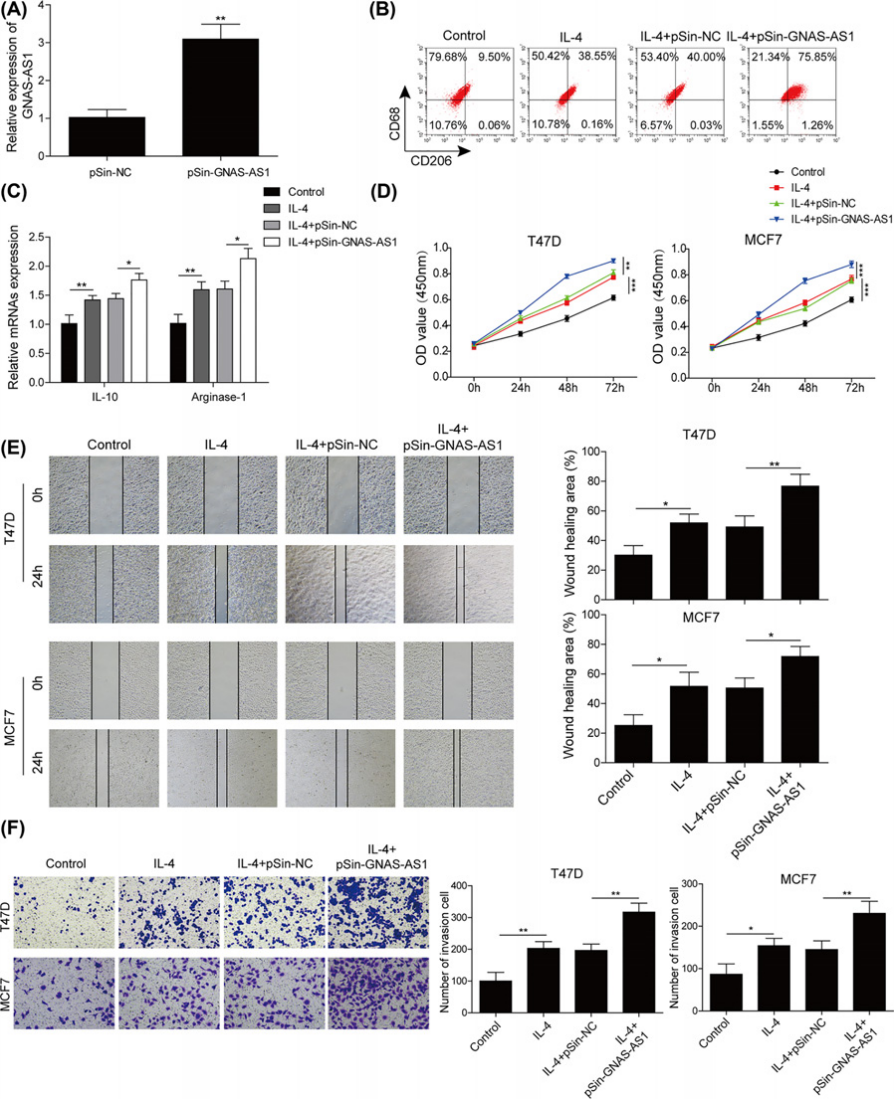
GNAS-AS1 facilitated M2 macrophage polarization and ER+ breast cancer cells proliferation and metastasis In IL-4 treated THP-1-differentiated macrophages, pSin-GNAS-AS1 plasmids or negative plasmids were transfected into cells, then next experiments were conducted. (**A**) The level of GNAS-AS1 was evaluated using qRT-PCR. (**B**) Flow cytometry was used to quantify the proportion of M1 or M2 macrophages. (**C**) qRT-PCR was performed to determine the expression levels of M2 macrophage markers (IL-10, Arginase-1). (**D**) CCK-8 assay was conducted to assess the cell viability of T47D and MCF-7 cells co-cultured with above treated THP-1-differentiated macrophages. (**E**) Wound healing assay was used to examine the migration of T47D and MCF-7 cells co-cultured with above treated THP-1-differentiated macrophages. (**F**) Transwell was applied to detect the invasion of T47D and MCF-7 cells co-cultured with above treated THP-1-differentiated macrophages. Data with error bars are presented as the mean ± SD. The Student’s *t* test and one-way ANOVA test were used to determine significance; *P<0.05, **P<0.01, ***P<0.001.

In order to further evaluate the roles of GNAS-AS1 in macrophage polarization and the progress of ER^+^ breast cancer cells, we established GNAS-AS1 overexpressing THP-1-differentiated macrophages by transfecting with pSin-GNAS-AS1 plasmids. Then, qRT-PCR analysis confirmed the increase of GNAS-AS1 expression in THP-1-diferentiated macrophages (Figure 2A). Next, cells were exposed to IL-4 to induce M2 macrophage polarization. Flow cytometry assay suggested that IL-4 stimulation markedly facilitated CD206^+^ cells proportion, which further enhanced by GNAS-AS1 overexpression (Figure 2B). Meanwhile, overexpressing GNAS-AS1 dramatically promoted the production of the M2 macrophage markers (IL-10, Arginase-1), compared with IL-4 treatment group (Figure 2C), indicating that GNAS-AS1 markedly promoted M2 macrophage polarization. We further detected the effects of GNAS-AS1 mediated M2-polarized macrophages on the proliferation, migration and invasion of T47D and MCF-7 cells. As shown in Figure 2D, IL-4 treatment significantly promoted cell proliferation, whereas it was further increased after GNAS-AS1 overexpression. Similarly, overexpression of GNAS-AS1 in IL-4 treated THP-1-differentiated macrophages also significantly enhanced cell migration and invasion (Figure 2E,F). These data showed that GNAS-AS1 mediated M2 macrophage polarization accelerated the capabilities of cell proliferation, migration and invasion of ER^+^ breast cancer cells.

### miR-433-3p was a target of GNAS-AS1

**Figure 3:**
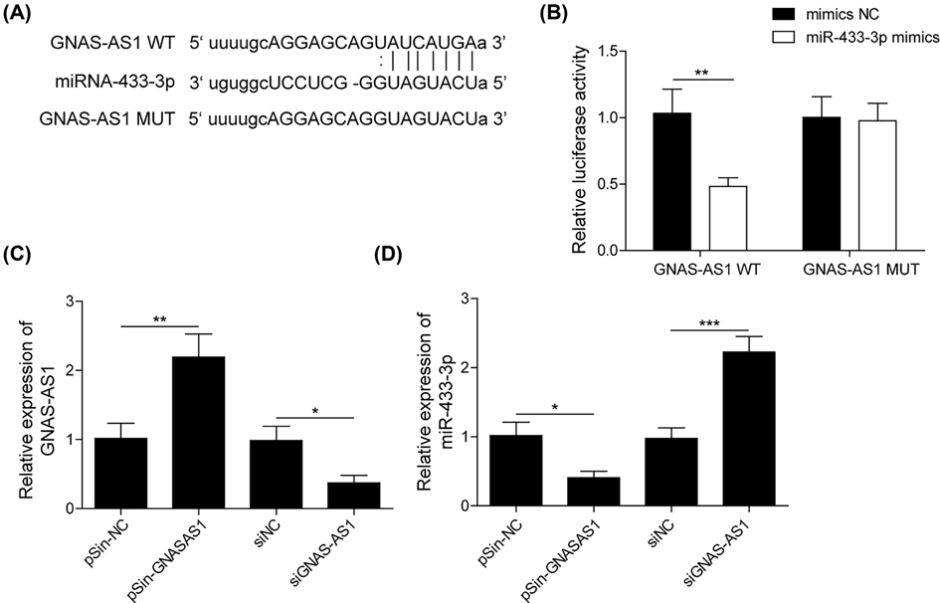
miR-433-3p was a target of GNAS-AS1 (**A**) The binding site of miR-433-3p on GNAS-AS1 3′-UTR region. (**B**) Dual luciferase reporter assay was used to validate the molecular relationship between miR-433-3p and GNAS-AS1 in THP-1-differentiated macrophages. (**C**) qRT-PCR was performed to assess the expression of GNAS-AS1 in THP-1-differentiated macrophages transfected with pSin-GNAS-AS1 plasmids or si-GNAS-AS1. (**D**) qRT-PCR was performed to assess the expression of miR-433-3p in THP-1-differentiated macrophages transfected with pSin-GNAS-AS1 plasmids or si-GNAS-AS1. Data with error bars are presented as the mean ± SD. The Student’s *t* test and one-way ANOVA test were used to determine significance; *P<0.05, **P<0.01, ***P<0.001.

LncRNA worked as a major regulator of miRNA to regulate gene expression on transcriptional and post-transcriptional level [20]. After prediction using Targetscan (http://www.targetscan.org), we found miR-433-3p might a potential target of GNAS-AS1 (Figure 3A). Hence, dual-luciferase reporter system was conducted to validate the interaction between GNAS-AS1 and miR-433-3p in THP-1 differentiated macrophages. As described in Figure 3B, compared with mimics group, miR-433-3p mimics significantly reduced the luciferase activity of THP-1-differentiated macrophages containing GNAS-AS1-WT plasmids, whereas no significant differ of luciferase activity was observed in THP-1-differentiated macrophages containing GNAS-AS1-MUT plasmids. qRT-PCR assay results showed that GNAS-AS1 overexpression by transfection with pSin-GNAS-AS1 plasmids obviously inhibited miR-433-3p expression. Conversely, GNAS-AS1 down-regulation by transfection with si-GNAS-AS1 markedly promoted miR-433-3p expression (Figure 3C,D). Taken together, these data demonstrated that miR-433-3p was a target of GNAS-AS1.

### miR-433-3p negatively affected the biological functions of GNAS-AS1

**Figure 4:**
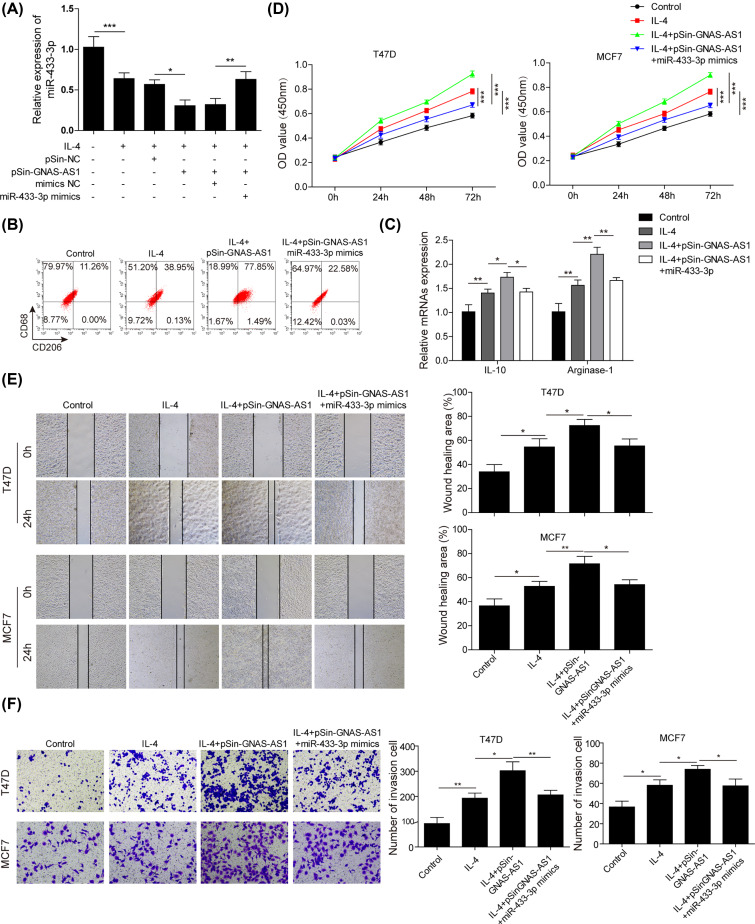
miR-433-3p negatively affected the biological functions of GNAS-AS1 In IL-4 treated THP-1-differentiated macrophages, pSin-GNAS-AS1 plasmids were co-transfected with/without miR-433-3p mimics, then next experiments were conducted. (**A**) qRT-PCR was performed to analyze the expression of miR-433-3p. (**B**) Flow cytometry was used to quantify the proportion of M1 or M2 macrophages. (**C**) qRT-PCR was applied to determine the expressions of M2 macrophage markers (IL-10, Arginase-1). (**D**) CCK-8 assay was conducted to assess the cell viability of T47D and MCF-7 cells co-cultured with above treated THP-1-differentiated macrophages. (**E**) Wound healing assay was used to detect the migration of T47D and MCF-7 cells co-cultured with above treated THP-1-differentiated macrophages. (**F**) Transwell assay was performed to elevate the invasion of T47D and MCF-7 cells co-cultured with above treated THP-1-differentiated macrophages. Data with error bars are presented as the mean ± SD. The Student’s *t* test and one-way ANOVA test were used to determine significance; *P<0.05, **P<0.01, ***P<0.001.

Next, we concentrated on the effects of miR-433-3p acted on GNAS-AS1 mediated biological roles. Firstly, Figure 4A was discribed that GNAS-AS1 up-regulation inhibited the expression of miR-433-3p, and this inhibitory effect was abolished when miR-433-3p mimics were co-transfected. Subsequently, As shown in Figure 4B,C, in IL-4 treatment THP-1-differentiated macrophages, miR-433-3p overexpression dramatically decreased the proportion of CD206^+^ macrophages and the expression levels of IL-10 and Arginase-1, compared with GNAS-AS1 overexpression, which suggested that miR-433-3p overexpression attenuated GNAS-AS1 mediated M2 macrophage polarization. Next, further functional experiments showed that GNAS-AS1 mediated M2-polarized macrophages markedly promoted the capabilities of cell proliferation, migration and invasion of T47D and MCF-7 cells, while these effects were reversed by miR-433-3p overexpression (Figure 4D–F). These finding indicated that miR-433-3p was negatively affected GNAS-AS1 functions on M2 macrophage polarization and ER^+^ breast cancer cell progression.

### miR-433-3p negatively regulated the expression of GATA3

**Figure 5:**
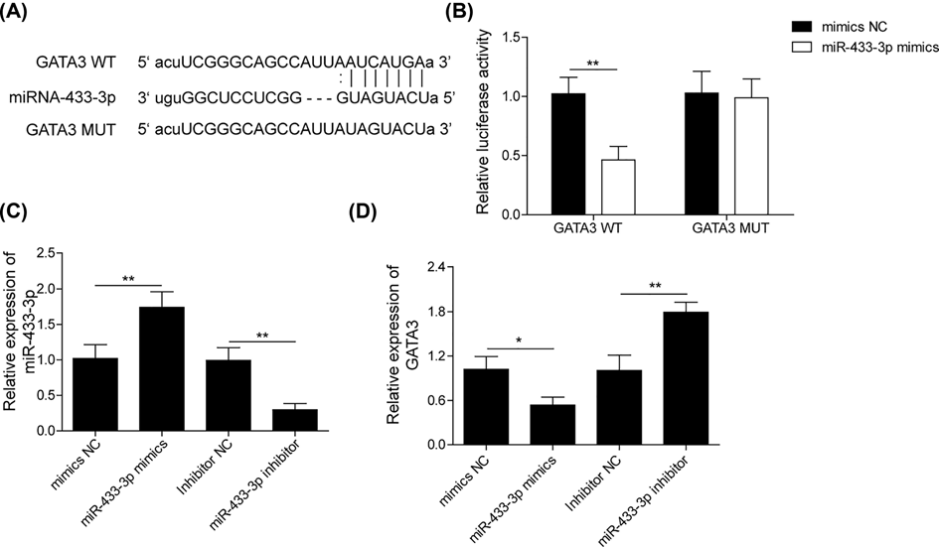
miR-433-3p negatively regulated the expression of GATA3 (**A**) The binding site of miR-433-3p on GATA3 3′-UTR region. (**B**) Dual luciferase reporter assay was used to validate the binding relationship between miR-433-3p and GATA3 in THP-1-differentiated macrophages. (**C**) qRT-PCR was performed to assess the expression of miR-433-3p in THP-1-differentiated macrophages transfected with miR-433-3p mimics or miR-433-3p inhibitor. (**D**) qRT-PCR was performed to assess the expression of GATA3 in THP-1-differentiated macrophages transfected with miR-433-3p mimics or miR-433-3p inhibitor. Data with error bars are presented as the mean ± SD; The Student’s *t* test and one-way ANOVA test were used to determine significance; *P<0.05, **P<0.01, ***P<0.001.

GATA3 is a transcriptional factor that plays crucial roles in regulation M2 macrophage polarization [21]. Predictive analysis (Targetscan) showed a potential binding site of miR-433-3p on the 3′UTR of GATA3 (Figure 5A). Dual luciferase reporter assay result was presented in Figure 5B, compared with mimics NC group, overexpressing miR-433-3p markedly decreased luciferase activity of THP-1-differentiated macrophages transfected with GATA3-WT plasmids, while had no effect in THP-1-differentiated macrophages transfected with GATA3-MUT plasmids. Subsequently, we next examined the regulatory patterns of miR-433-3p on GATA3 expression. As showed in Figure 5C,D, the up-regulation of miR-433-3p by transfected with miR-433-3p mimics greatly suppressed GATA3 expression, while down-regulation of miR-433-3p caused by miR-433-3p inhibitor elevated GATA3 expression. The data from above-mentioned demonstrated that miR-433-3p directly inhibited the expression of GATA3.

### Silencing of GATA3 abolished the biological roles of GNAS-AS1

**Figure 6:**
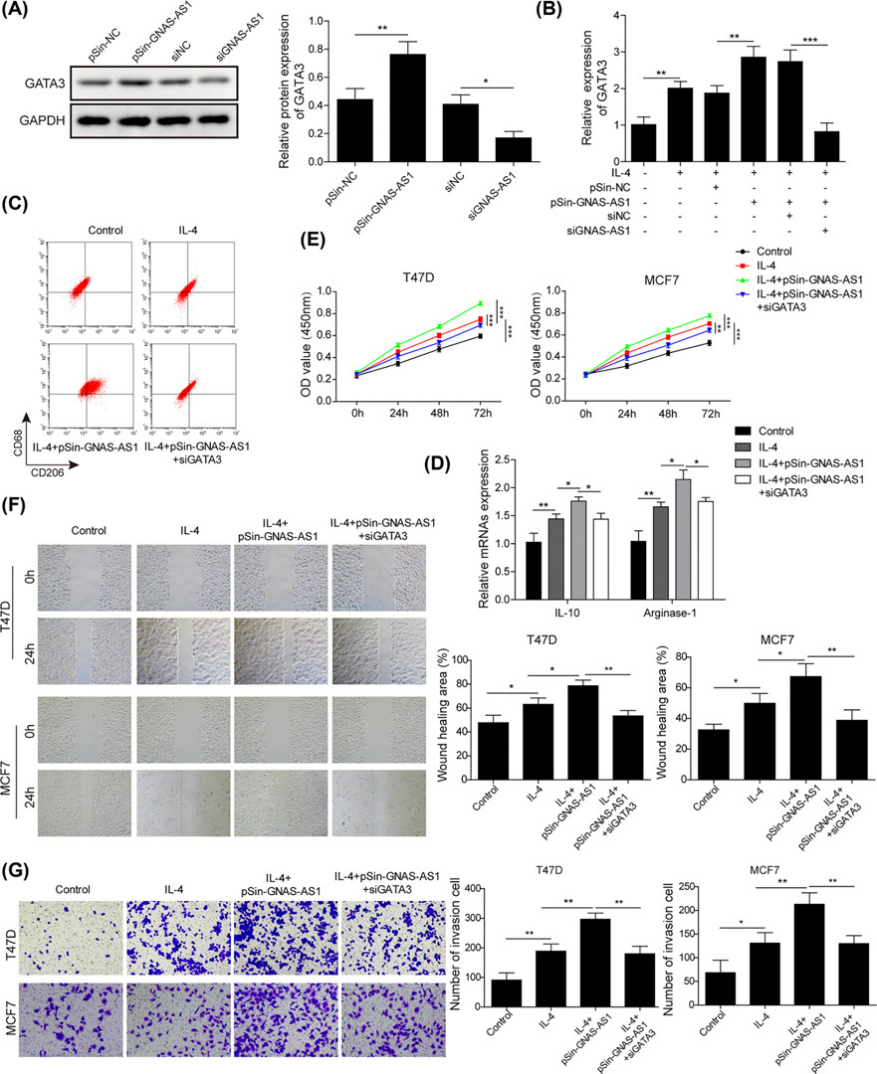
Silencing of GATA3 abolished the biological roles of GNAS-AS1 (**A**) The protein level of GATA3 was examined by Western blot analysis in THP-1 cells transfected with pSin-GNAS-AS1 plasmids or si-GNAS-AS1. (**B–G**) In IL-4 treated THP-1-differentiated macrophages, pSin-GNAS-AS1 plasmids were co-transfected with/without siGATA3, then, next experiments were performed. (B) The mRNA level of GATA3 was analyzed by qRT-PCR. (C) Flow cytometry was used to quantify the proportion of M2 macrophages polarization. (D) qRT-PCR was performed to determine the expressions of M2 macrophage markers (IL-10, Arginase-1). (E) CCK-8 was used to detect the migration of T47D and MCF-7 cells co-cultured with above treated THP-1-differentiated macrophages. (F) Wound healing assay was used to detect the migration of T47D and MCF-7 cells co-cultured with above treated THP-1-differentiated macrophages. (G) Transwell was used to detect the invasion of T47D and MCF-7 cells co-cultured with above treated THP-1-differentiated macrophages. Data with error bars are presented as the mean ± SD. The Student’s *t* test and one-way ANOVA test were used to determine significance; *P<0.05, **P<0.01, ***P<0.001.

On previous basis in this work, we next attempted to understand the association between GATA3 and GNAS-AS1. Western blot assay was performed in THP-1-differentiated macrophages revealed that the protein level of GATA3 was markedly increased by GNAS-AS1 overexpression, whereas it was decreased by GNAS-AS1 silence (Figure 6A). Next, qRT-PCR assay showed that GATA3 expression were increased after IL-4 treatment, and its further enhanced by GNAS-AS1 overexpression, while the effect induced by GNAS-AS1 overexpression was markedly weakened by si-GATA3 transfection (Figure 6B). Subsequently, flow cytometry was applied to explore whether GATA3 was involved in M2 macrophage polarization mediated by GNAS-AS1. As shown in Figure 6C,D, in IL-4 treated THP-1-differentiated macrophages, knockdown of GATA3 obviously reversed the roles of GNAS-AS1 on the expressions of M2 macrophage markers, including CD206, IL-10 and Arginase-1, suggesting that knockdown of GATA3 impeded GNAS-AS1 mediated M2 macrophage polarization. Moreover, GNAS-AS1 overexpressed THP-1-differentiated macrophages mediated the promotion on the capabilities of proliferation, migration and invasion of T47D and MCF-7 cells were significantly diminished by GATA3 down-regulation (Figure 6E–G). Thus, these results suggested that GATA3 was a downstream target of GNAS-AS1 and involved in the regulatory network of GNAS-AS1 on M2 macrophage polarization and ER^+^ breast cancer cells progression.

## Discussion

As one of the most common cancer in women, breast cancer accounts for 25.1% of all cancers [22]. Based on the pathological phenotype, breast cancer is categorized into four main intrinsic molecular subtypes, human epidermal growth factor receptor 2 (HER2) enriched, basal-like, luminal A and luminal B [23]. Accumulating evidences have demonstrated that macrophage polarization is closely associated with the initiation and development of cancer [24]. Yin et al. showed that the macrophage polarization stimulated by breast cancer cell derived-exosomes was closely connected to with lymph node metastasis [25]. Another report focused on TAMs roles in breast cancer proposed that M2 macrophages enhanced cell proliferation and invasion through recruiting immunosuppressive leukocytes, remodeling the extracellular matrix (ECM) and stimulating angiogenesis [26]. In addition, the macrophage polarization status was identified to be rapidly induced or re-polarized by complex endogenous cellular signaling pathways and multiple regulators, such as lncRNAs and miRNAs [7,27,28]. In our study, we have demonstrated that GNAS-AS1 overexpression accelerated M2 macrophage polarization, and thus promoting the capabilities of proliferation, migration and invasion in ER^+^ breast cancer cells, which may be a novel molecular mechanism of ER^+^ breast cancer development.

LncRNAs are endogenous transcripts and play important roles in various biological and pathogenic processes through functional interaction with DNA, RNA and protein [29]. Recently, emerging reports have showed that lncRNAs were a class of key regulator of TAMs. Ji et al. identified that lncRNA-MM2P promoted the polarization of M2 macrophages derived by cytokines and accelerated M2 macrophages mediated angiogenic feature through elevating phosphorylation on STAT6 [28]. Moreover, GNAS-AS1 was confirmed to promote M2 polarization of macrophages in NSCLC, which further promoted the development of the malignant tumor [15]. Similarly, we found GNAS-AS1 was high expressed in ER^+^ breast cancer tissues, cell lines and M2 macrophages. Meanwhile, GNAS-AS1 overexpression markedly enhanced the proportion of M2-polarized macrophages, which obviously accelerated the proliferation, migration and invasion of ER^+^ breast cancer cells, suggesting the potential application of GNAS-AS1 in the regulation of TME and breast cancer progression tumor as a therapeutic target.

miRNAs are small endogenous RNA molecules which post-transcriptionally silence gene expression in both biological and pathogenic contexts. Previous studies demonstrated that miR-433 was low expressed and exerted anti-tumor effects in different neoplasms [30]. Moreover, miR-433-3p suppressed hematopoietic cell growth and differentiation in myeloproliferative neoplasms, while promoted osteoblast differentiation during bone formation, suggesting the roles of miR-433-3p on tumor development and cell differentiation [31,32]. In our study, we first indicated that miR-433-3p was a target of GNAS-AS1, and involved in the regulation of GNAS-AS1 mediated M2 macrophage polarization and ER^+^ breast cancer progression. Furthermore, miR-433-3p directly targeted GATA 3′-UTR to suppress its expression in THP-1-differentiated macrophages. These finding indicated a new insight of miR-433-3p in the pathogenesis of ER^+^ breast cancer.

A number of researches showed that the overexpression of estrogen is an important factor leading to the excessive proliferation and even canceration of breast epithelial cells, and the expression level of estrogen and its receptor is clinically positively correlated with the expression of GATA3 [33,34]. GATA3, a member of the GATA transcription factor family, plays important roles in regulating breast differentiation and immune system regulation, and with a differ on the prognosis and function on different subtypes of breast cancer, such as basal-like breast cancer and luminal breast cancer [35]. As previous reported, GATA3 could activate the transcription of estrogen receptor by serving as a transcriptional co-activator to interact with KDM4B or ASH2L [36,37]. Meanwhile, another study focused on luminal breast cancer have revealed that GATA3 is frequently mutated and its levels are significantly elevated, and could mediate the transformation of normal cells into breast cancer through deregulation of BCL2, DACH1 and THSD4 [35]. In addition, GATA3 has also been proved to promote the differentiation of T cells into Th2 cells by increasing cell effector Th2, thus affecting the immune response of tumor cells [38]. Likewise, GATA3 also promoted the proliferation and invasion of high-serous ovarian cancer cells by regulating M2-type macrophage polarization [39]. Similar results were observed in our study, GATA3, worked as a downstream target of GNAS-AS1/miR-433-3p axis, was identified to promote the M2 polarization of macrophages and enhance the capabilities of proliferation, migration and invasion of ER^+^ breast cancer cells. Therefore, we speculated that GATA3 may play a promoting role on the occurrence of ER^+^ breast cancer by regulating the TME and ER transcription.

In conclusion, our studies revealed that GNAS-AS1 was up-regulated in M2 macrophages and ER^+^ breast cancer. Importantly, we proposed a new modulator mechanism that GNAS-AS1 promoted proliferation, migration and invasion of ER^+^ breast cancer cells by inducing M2 macrophage polarization via regulating miR-433-3p/GATA3 axis, which may provide an effective strategy for breast cancer treatment.

## Abbreviations

CCK-8: Cell Counting Kit-8
ECM: extracellular matrix
FBS: fetal bovine serum
GATA3: GATA-binding protein 3
GNAS-AS1: GNAS antisense
HER2: human epidermal growth factor receptor 2
IFN: interferon
IL-10: interleukin-10
LncRNA: long non-coding RNA
LPS: lipopolysaccharides
NSCLC: non-small cell lung cancer
PBMC: peripheral blood mononuclear cell
qRT-PCR: real-time quantitative PCR
TAM: tumor-associated macrophages
TGF-β: transforming growth factor-β
TME: tumor microenvironment
